# TorchMD-Net 2.0: Fast Neural Network Potentials for Molecular Simulations

**Published:** 2024-02-27

**Authors:** Raul P. Pelaez, Guillem Simeon, Raimondas Galvelis, Antonio Mirarchi, Peter Eastman, Stefan Doerr, Philipp Thölke, Thomas E. Markland, Gianni De Fabritiis

**Affiliations:** † Computational Science Laboratory, Universitat Pompeu Fabra, Barcelona Biomedical Research Park (PRBB), C Dr. Aiguader 88, 08003 Barcelona, Spain.; ‡ Acellera Labs, C Dr Trueta 183, 08005, Barcelona, Spain; ¶ Department of Chemistry, Stanford University, Stanford, CA 94305, USA; § Institució Catalana de Recerca i Estudis Avançats (ICREA), Passeig Lluis Companys 23, 08010 Barcelona, Spain

## Abstract

Achieving a balance between computational speed, prediction accuracy, and universal applicability in molecular simulations has been a persistent challenge. This paper presents substantial advancements in the TorchMD-Net software, a pivotal step forward in the shift from conventional force fields to neural network-based potentials. The evolution of TorchMD-Net into a more comprehensive and versatile framework is highlighted, incorporating cutting-edge architectures such as TensorNet. This transformation is achieved through a modular design approach, encouraging customized applications within the scientific community. The most notable enhancement is a significant improvement in computational efficiency, achieving a very remarkable acceleration in the computation of energy and forces for TensorNet models, with performance gains ranging from 2-fold to 10-fold over previous iterations. Other enhancements include highly optimized neighbor search algorithms that support periodic boundary conditions and the smooth integration with existing molecular dynamics frameworks. Additionally, the updated version introduces the capability to integrate physical priors, further enriching its application spectrum and utility in research. The software is available at https://github.com/torchmd/torchmd-net.

## 1 Introduction

Neural Network Potentials (NNPs)^1–7^ are emerging as a key approach in molecular simulations, striving to optimize the balance between computational efficiency, predictive accuracy, and generality.

Some software frameworks to facilitate the use of neural network potentials have been developed, such as SchNetPack,^8^ TorchANI,^9^ DeePMD-Kit,^10^ and others. Among the first to appear, we released TorchMD-Net, initially designed for the Equivariant Transformer architecture^11^ and a graph network, a simpler invariant graph neural network tailored for neural network potentials for protein coarse-graining.^12^ Over time, TorchMD-Net has expanded its model architectures to include TensorNet,^13^ an *O*(3)-equivariant message-passing neural network utilizing rank-2 Cartesian tensor representations which achieved state-of-the-art accuracy on benchmark datasets. This evolution positions TorchMD-Net not just as a standalone tool, but as a versatile library for the development of NNPs.

Efficiency has been at the forefront of recent enhancements to TorchMD-Net. Among the optimizations, CUDA graphs have been integrated, providing a performance boost especially for smaller workloads. TorchMD-Net has also incorporated the latest versions of its key dependencies (mainly PyTorch^14^ and PyTorch Lightning^15^), with a notable addition being the search for compatibility with the torch.compile submodule from PyTorch 2.0, a feature that compiles Just-In-Time (JIT) the modules into optimized kernels. While TorchMD-Net has introduced low precision modes (i.e. bfloat16) primarily as an exploratory tool for researchers, high precision (float64) is also available for ensuring detailed correctness checks during prototyping.

The new technical enhancements include the introduction of periodic boundary conditions, a CUDA-optimized neighbor list, and memory-efficient dataset loaders. The inclusion of TorchMD-Net in the conda-forge^16^ package repository and the release of the documentation^17^ are steps taken to enhance its accessibility to researchers. Another feature is TorchMD-Net’s capacity to blend empirical physical knowledge into NNPs via priors. The integration of atom-wise and molecule-wise priors, such as the Ziegler-Biersack-Littmark^18^ and Coulomb potentials, allows for a more nuanced approach in simulations.

TorchMD-Net emphasizes compatibility with leading molecular dynamics (MD) packages, especially with OpenMM.^19^ OpenMM, widely recognized in the computational chemistry field, can now interface directly with TorchMD-Net through the OpenMM-Torch^20^ plugin. This integration has been a collaborative effort, with OpenMM-Torch being co-developed by the core teams of both OpenMM and TorchMD-Net. This ensures streamlined and effective utilization of TorchMD-Net models within OpenMM’s simulation framework.

In the following sections we provide an overview of the TorchMD-Net framework. The manuscript is ordered as follows. In the Methods section we go over the currently available NNP architectures. We continue in section Training with details about the different parts involved in the training and deployment of these architectures and how they are exposed in TorchMD-Net. Then, in section Optimization, we lay out the optimization strategies employed in this release. Finally, we present a series of validation and performance results in the Results section.

TorchMD-Net is freely available with a permissive licence (MIT) at https://github.com/torchmd/torchmd-net.

## 2 Methods

We interpret a neural network potential as a machine learning model that takes as input a series of atomic positions, denoted by R, embedding indices such as atomic numbers, Z, and optionally charges (which might be per-sample or per-atom), q, and outputs a per-sample scalar value and optionally its negative gradient with respect to the positions, typically interpreted as the potential energy and atomic forces, respectively. Note, however, that TorchMD-Net is not limited to this interpretation of the outputs, which are generally labelled as y and neg_dy respectively.

**Figure 1:**
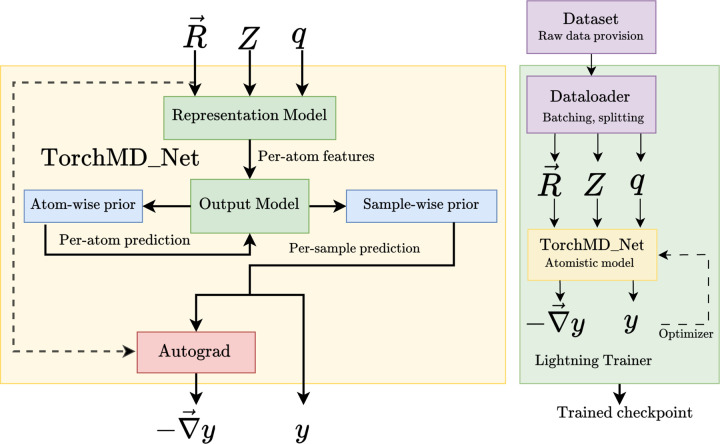
The main module in TorchMD-Net is called TorchMD_Net from the torchmdnet.models.model module. This class combines a given representation model (such as the Equivariant Transformer), an output model (such as the scalar output module) and a prior model (such as the Atomref prior), producing a module that takes as input a series of atoms features and outputs a scalar value (i.e energy per molecule) and when derivative = True, its negative gradient with respect to the input positions (i.e atomic forces).

Figure 1 provides a comprehensive overview of the TorchMD-Net architecture. The diagram’s left section illustrates the various components of the primary module, designated as TorchMD_Net, which constitutes, conceptually and in the API itself, a NNP model. Each component within this object is modular and customizable, allowing for the creation of diverse models. At the heart of the NNP is the representation_model. This part of the architecture takes the set of inputs stated above and outputs a series of per-atom features. These features are subsequently fed into an output_model. The purpose of this model is to further process these features into single atomic values, which typically will be aggregated and will represent the total potential energy, though it can represent other per-sample or per-atom quantities as well, depending on the specifics of its design and (optional) aggregation scheme. Output models normally include learnable parameters (e.g. a multilayer perceptron). Prior models can be employed to augment either the atom-level features or the aggregated per-molecule value with further physical insights. Furthermore, the framework integrates PyTorch’s Autograd for automatic differentiation, enabling the computation of the negative gradient of the per-molecule scalar prediction with respect to atomic positions. This is particularly relevant when interpreting the per-molecule value as the potential energy, as it yields the atomic forces in a way that ensures, by construction, that the resulting force field is energy conserving.

This modular logic allows for flexibility in the combination of representation models and output models. Therefore, by building a custom output module, researchers can make use of the representation models for other prediction tasks beyond potential energy and forces.

### 2.1 Available representation models

Current models in TorchMD-Net at the time of writing are message-passing neural networks^21,22^ (MPNNs) which learn approximations to the many-body potential energy function. Atoms are identified with graph nodes embedded in 3D space, building edges between them after the definition of some cutoff radius. The neural network uses atomic and geometric information to learn expressive representations by propagating, aggregating, and transforming features from neighboring nodes found within the cutoff radius.^23,24^ In most current NNPs, after several message passing steps, node features are used to predict per-atom scalar quantities which are identified with atomic contributions to the energy of the molecule.

#### 2.1.1 New architecture: TensorNet

TensorNet^13^ is an *O*(3)-equivariant model based on rank-2 Cartesian tensor representations. Euclidean neural network potentials^25–27^ have been shown to achieve state-of-the-art performance and better data efficiency than previous models, relying on higher-rank equivariant features which are irreducible representations of the rotation group, in the form of spherical tensors. However, the computation of tensor products in these models can be computationally demanding. In contrast, TensorNet exploits the use of Cartesian rank-2 tensors (3×3 matrices) which can be very efficiently decomposed into scalar, vector and rank-2 tensor features. Furthermore, Clebsch-Gordan tensor products are substituted by straightforward and node-level 3×3 matrix products. Overall, these properties allow TensorNet to achieve state-of-the-art accuracy on common benchmark datasets with a reduced number of message-passing steps, learnable parameters, and computational cost. The prediction of up to rank-2 molecular properties that behave appropriately under geometric transformations such as reflections and rotations is also possible.

#### 2.1.2 Equivariant Transformer

The Equivariant Transformer^11^ (ET) is an equivariant neural network that uses both scalar and Cartesian vector representations. The distinctive feature of the ET in comparison to other Cartesian vector models such as PaiNN^28^ or EGNN^29^ is the use of a distance-dependent dot product attention mechanism, which achieved state-of-the-art performance on benchmark datasets at the time of publication. Furthermore, the analysis of attention weights allowed us to extract insights into the interaction of different atomic species for the prediction of molecular energies and forces. The model also exhibits a low computational cost for inference and training in comparison to some of the most used NNPs in the literature.^30^

As part of the current release, we removed a discontinuity at the cutoff radius. In the original description, vector features’ residual updates, as opposed to scalar features’ updates, received contributions from the value pathway of the attention mechanism which were not being properly weighted by the cosine cutoff function envelope, which is reflected in Eq. 9 in the original paper.^11^ We fixed it by applying $\phi \left(d_{ij}\right)$, i.e., $\text{split}\left(V_{j}⊙D_{ij}^{V}\right)\to \text{split}\left(\phi \left(d_{ij}\right)V_{j}⊙D_{ij}^{V}\right)$. To ensure backward compatibility, this modification is only applied when setting the new ET argument vector_cutoff = True. The impact of this modification is evaluated in the results section.

#### 2.1.3 Graph Network

The graph network is an invariant model inspired by both the SchNet^31^ and PhysNet^32^ architectures. The network was optimized to have satisfactory performance on coarse-grained proteins, allowing the building of NNPs that correctly reproduce fast-folder protein free energy landscapes.^12^ In contrast to the ET and TensorNet, the graph network only uses relative distances between atoms as geometrical information, which are invariant to translations, rotations, and reflections. The distances are used by the model to learn a set of continuous filters that are applied to feature graph convolutions as in SchNet,^31^ progressively updating the initial atomic embeddings by means of residual connections.

### 2.2 Prior models

Priors are additional physical terms that can be introduced for the prediction of potential energies. Some of these terms have been used in NNPs in the literature,^33,34^ sometimes even including learnable parameters. In TorchMD-Net, we provide some predefined priors, which can be optionally added to the neural network prediction:

- Atomref: These are per-element atomic reference energies, which are usually provided directly in the dataset. In this case, the neural network has to predict the remaining contribution to the potential energy, which can be regarded as the formation energy of the molecule. There is also the option of making this prior learnable, in which case it is initialized with atomic reference energies, but these contributions are modified during training.
- Coulomb: This prior corresponds to the usual Coulomb electrostatic interaction, scaled by a cosine switching function to reduce its effect at short distances. Using this prior requires providing per-atom partial charges.
- D2 dispersion: In this case, the prior corresponds to the D2 dispersive correction used in DFT-D2.^35^ *C*_6_ coefficients and Van der Waals radii for elements are already incorporated in the method.
- ZBL potential: This prior implements the Ziegler-Biersack-Littmark (ZBL) potential for screened nuclear repulsion as described in Ref 18. It is an empirical potential effectively describing the repulsion between atoms at very short distances, and only atomic numbers need to be provided.

Note that forces are computed directly by autograd when adding the energy contributions coming from the priors before the backward automatic differentiation step. Even though the previous terms are the currently predefined options in TorchMD-Net, all these priors are derived from a general BasePrior class, which easily allows researchers to implement their own priors, following the modular logic behind the framework.

### 2.3 Training

The right diagram in Figure 1 depicts the main training loop in TorchMD-Net. A Dataset provides sample/output pairs for the NNP and is divided into training, validation and testing sets and batched by a Dataloader (as provided by the Pytorch Geometric library^36^). We make use of the PyTorch Lightning library’s^15^ trainer, which also allows multi-GPU training. Checkpoints are generated during training, containing the current weights of the model, which can then be subsequently loaded for inference or further training.

#### 2.3.1 Datasets

Within TorchMD-Net, datasets can be accessed through the YAML configuration file for use with the torchmd-train utility or programmatically via the Python API. Predefined datasets include SPICE,^37^ QM9,^38^ WaterBox,^39^ (r)MD17^40^,^41^ MD22,^42^ ANI1,^43^ ANI1x,^44^ ANI1ccx,^44^ ANI2x^45^ and the COMP6^46^ evaluation dataset with all its subsets (ANIMD, DrugBank, GDB07to09, GDB10to13, Tripeptides and S66X8), offering diverse training environments for molecular dynamics and quantum chemistry applications. These datasets serve as common benchmarks in the field of neural network potentials. However, on top of these, the framework allows the flexible incorporation of user-generated datasets for customized applications. The Custom dataset functionality allows users to train models with molecular data encapsulated in simple NumPy file formats without writing a single line of code. By specifying paths to coordinate and embedding index (e.g. atomic numbers) and reference energy and force files, researchers can easily integrate their datasets into the training process. This capability ensures TorchMD-Net’s adaptability to a wider array of applications beyond its pre-packaged offerings. In addition, TorchMD-Net offers support for other popular dataset formats, such as HDF5. Special care is taken to ensure data is cached as much as possible, using techniques such as in memory datasets and memory mapped files.

#### 2.3.2 Losses

During training, a weighted sum of mean squared error (MSE) losses of energy and forces is used, weighting each of them according to user input. In validation, we provide both L1 and MSE losses separately for energies and forces, while for testing L1 losses alone are used. The framework allows to use an exponential moving average (EMA) to update the losses during the training and validation stages to smooth out progression of loss values.

### 2.4 Usage examples

In the following sections we showcase code for some typical usecases of TorchMD-Net. While these snippets are generally self-contained the reader is pointed to the online documentation^17^ for further information.

#### 2.4.1 Training code example

The project introduces a command line tool, torchmd-train, designed as a code-free method for model training. This tool is set up through a YAML file, with several examples available in the TorchMD-Net GitHub repository for reference. However, we also offer an illustrative script here that outlines the process of training an existing model using the Python API. The LNNP class, found within the torchmdnet.module module, encapsulates the procedures for both the creation and training of a model. This class is inherited from Pytorch Lightning LightningModule, offering all the extensive customization available in it. The following is a succinct yet comprehensive example of how to utilize LNNP for training purposes:

**Table:** 

1	from torchmdnet.data import DataModule
2	from torchmdnet.module import LNNP
3	from pytorch_lightning import Trainer
4	args = {
5	’dataset’: ’ANI1X’,
6	’model’: ’tensornet’,
7	’num_epochs’: 200,
8	’embedding_dimension’: 128,
9	’num_layers’: 2,
10	’num_rbf’: 32,
11	’rbf_type’: ’expnorm’,
12	’trainable_rbf’: False,
13	’activation’: ’silu’,
14	’cutoff_lower’: 0.0,
15	’cutoff_upper’: 5.0,
16	’max_z’: 100,
17	’max_num_neighbors’: 64,
18	’derivative’: True # So the model returns forces.
19	}
20	data = Data Module(args)
21	data.prepare_data()
22	data.setup("fit")
23	lnnp = LNNP(args,
24	prior_model = None,
25	mean = data.mean,
26	std = data.std)
27	trainer = Trainer(max_epochs = args [’num_epochs’])
28	trainer.fit(lnnp, data)
29	model = LNNP.load_from_checkpoint(trainer.checkpoint_callback.best_model_path)
30	trainer = pl.Trainer(inference_mode = False)
31	trainer.test(model, data)

This example shows the minimal steps required to prepare data, initialize the LNNP class, train and test a model using PyTorch Lightning’s Trainer. The Trainer here is simplified for brevity; in practice, additional callbacks and logger configurations could be added.

#### 2.4.2 Loading a Trained Model for Inference

After training a model, the next logical step is to use it for inference. TorchMD-Net offers a dedicated function, load_model, to facilitate this. Below is a concise example:

**Table:** 

1	from torchmdnet.models.model import load_model
2	# Define the path to the saved model checkpoint
3	checkpoint_path = "path / to/ saved_model_checkpoint.ckpt"
4	# Load the model
5	loaded_model = load_model (checkpoint_path)
6	# Prepare the input data (atomic numbers, positions, batch index, etc.)
7	# For demonstration, these are placeholders and should be replaced with actual data
8	input_data = {
9	’z’: torch.Tensor ([…]),
10	’pos’: torch.Tensor ([…]),
11	’batch’: torch.Tensor ([…])
12	# … other optional fields
13	}
14	# Perform inference
15	energy, forces = loaded_model (**input_data)
16	# Energy and forces are now available for further analysis or visualization

In this example, checkpoint_path should point to the location where the trained model checkpoint is saved. The input_data dictionary should be populated with the actual atomic numbers, positions, and other required or optional fields. Finally, energy and forces are obtained from the loaded model and can be used as needed.

#### 2.4.3 Integration with OpenMM

It is possible to run TorchMD-Net neural network potentials as force fields in OpenMM^19^ to run molecular dynamics. The OpenMM-Torch^20^ package is leveraged for this. Integration consists of writing a wrapper class to accommodate for the unit requirements of OpenMM and to provide to the model any information not proper to OpenMM (like the embedding indices). The following code showcases an example on how to add a TorchMD-Net NNP as an OpenMM Force.

**Table:** 

1	from torchmdnet.models.model import load_model
2	from openmmtorch import Torch Force
3	from openmm import System
4	
5	class Wrapper (torch.nn. Module):
6	
7	def __init__(self, embeddings, model):
8	super (Wrapper, self). __init__()
9	self.embeddings = embeddings
10	# Open MM will compute the forces
11	# by backpropagating the energy,
12	# so we can load the model with derivative = False
13	self.model = load_model (model, derivative = False)
14	
15	def forward (self, positions):
16	# Open MM works with nanometer positions
17	# and kilojoule per mole energies
18	# Depending on the model, you might need
19	# to convert the units
20	positions = positions * 10.0 # nm -> A
21	energy = self.model (z= self.embeddings, pos = positions) [0]
22	return energy * 96. 4916 # eV -> kJ/ mol
23	
24	# The embeddings used during training (e. g. atomic numbers)
25	# for each atom in the simulation.
26	z = torch.tensor([1,1], torch.long)
27	model = torch.jit.script(Wrapper(z, "model.ckpt"))
28	# Create a Torch Force object from the model
29	torch_force = openmmtorch.Torch Force(model)
30	system = System()
31	# The Torch Force object can be used as a regular Open MM Force
32	system.add Force(torch_force)
33	# Set up the rest of the Open MM simulation
34	# …

### 2.5 Optimization techniques

Typical neural network potential (NNP) algorithms implemented in PyTorch^14^ comprise a series of sequential operations such as multilayer perceptrons and message passing operations.

As PyTorch operations translate into highly optimized CUDA kernel calls, the efficiency of modern GPUs often turns kernel launching overhead into a performance bottleneck. CUDA graphs address this by consolidating multiple kernel calls into a single graph, drastically reducing kernel launch overhead. However, CUDA graphs impose stringent limitations. These include the need for static shapes in graphed code sections, which can lead to costly recompilations or memory inefficiencies, and the exclusion of operations requiring CPU-GPU synchronization.

Conversely, developments in the compiler community^47^, including technologies like OpenAI’s Triton^48^ and subsequently PyTorch enhancements, are gradually diminishing the reliance on CUDA graphs by automatically changing the structure of the code in ever more profound ways (i.e kernel fusion^49–51^). These advancements, such as TorchDynamo introduced in PyTorch 2.0 through torch.compile, optimize code structure through Just-In-Time (JIT) compilation.

Even with JIT, and in general transpilation-based techniques, CUDA graphs often provide the best out-of-the-box performance improvements and at the bare minimum, facilitate the optimizations introduced by the former. Encapsulating a piece of code within a CUDA graph, a process known as ‘stream capture’, necessitates adherence to several specific requirements. This often demands substantial modifications to the code. Crucially, for code to be eligible for capture, it must avoid any CPU-GPU synchronization activities, including synchronization barriers and memory copies. Additionally, all arrays involved in the operations must possess static shapes and fixed memory addresses, precluding any dynamic memory allocations during the process.

The CUDA graph interface in PyTorch alleviates many challenges associated with adapting code for stream capture. It particularly excels in managing memory allocations within captured environments automatically and transparently. However, challenges arise in specific implementations, as exemplified by TensorNet. The main issue in TensorNet is its neighbor list, which inherently varies in shape at each inference step due to the fluctuating number of neighbors. This variation affects the early stages of the architecture, resulting in TensorNet primarily operating on dynamically shaped tensors. To address this, we implemented a static shape mode that creates placeholder neighbor pairs up to a predetermined maximum. We then ensure the architecture disregards these placeholders’ contributions. Although this method increases the initial workload, our empirical data indicates that the performance gains from capturing the entire network substantially outweigh this added overhead.

In the following sections, we explore the impact of these optimizations on both inference and training performance.

#### 2.5.1 Neighbor search and periodic boundary conditions

**Figure 2:**
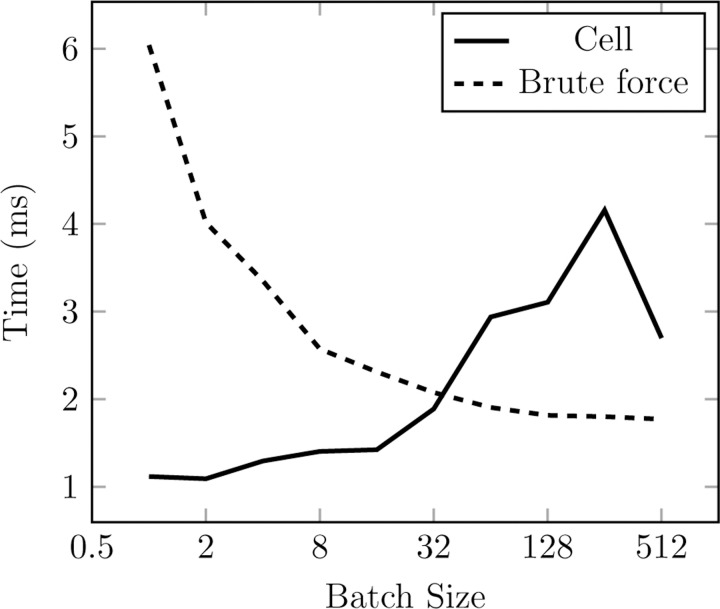
Performance comparison of cell (solid line) and brute-force (dashed line) neighbor search strategies across different batch sizes for a random cloud of 32k particles with 64 neighbors per particle on average. The particles are split into a certain number of batches.

**Figure 3:**
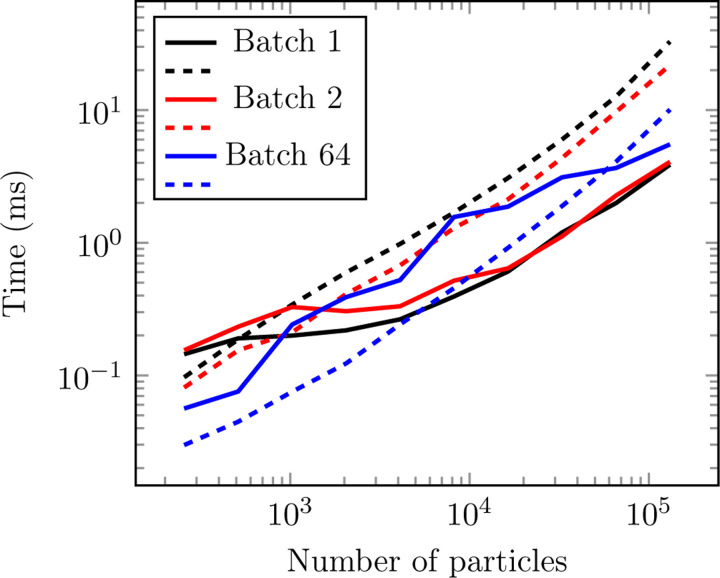
Performance comparison of cell (solid line) and brute-force (dashed line) neighbor search strategies across different batch sizes for a random cloud of particles with 64 neighbors per particle on average. Cell list performance tends to degrade with increasing batch size, while the opposite is true for brute force.

Message-passing neural networks, such as the architectures currently supported in the framework, require a list of connections among nodes referred to as edges. This list is constructed by proximity after the definition of a cutoff radius (a neighbor list). TorchMD-Net offers a neighbor list construction engine specifically tailored for NNPs, exposing a naive brute-force *O*(*N* ^2^) algorithm that works best for small workloads and a cell list (a standard *O*(*N* ) hash-and-sort strategy widely used in MD^52,53^) that performs better for large systems (see Figures 3 and 2). Effectively, this engine makes neighbor search a negligible part of the overall computation.

Special measures are taken into account to ensure that the neighbor search is compatible with CUDA-graphs. For this matter, it is required that the neighbor search works on a statically-shaped set of input/outputs, which poses a problem given that the number of neighbor pairs in the system is not known in advance and is bound to change from input to input. We solve this by requiring an upper bound for the number of pairs in the system and padding the outputs with a special value (−1) for unused pairs. Furthermore, TorchMD-Net architectures support rectangular and triclinic periodic boundary conditions.

Contrary to usual MD workloads, it is common to have batches of input samples in NNPs. This owns to the very nature of neural network training but also can benefit inference (for instance, allowing the possibility of running many simulations in parallel, like TorchMD^54^ does). Our neighbor list is able to handle arbitrary batch sizes while maintaining compatibility with CUDA graphs. The current cell list implementation constructs a single cell list including atoms for all batches, excluding pairs of particles that pertain to different batches when finding neighbors in it. This makes it so that each particle has to perform a check against every other particle in the vicinity for all batches, which degrades performance with increasing batch size. We find this to be an acceptable compromise given that doing it this way facilitates compatibility with CUDA graphs and we assume that with increasing number of particles (where the cell list excels) the typical batch size will decrease. Still, the particularities of the cell list implementation makes its performance specially susceptible to the batch size, as evidenced by the variability observed in the cell list curves in figures 3 and 2.

All data presented in this section was gathered in an RTX4090 NVIDIA GPU using CUDA 12. Each point is obtained by averaging 50 identical executions. Warmup executions are also performed before measuring.

#### 2.5.2 Training

Optimizing neural network training presents distinct challenges compared to inference optimization. Primarily, the variable length of each training sample, exacerbated by batching processes (where a varying number of samples constitute a single network input), impedes optimizations dependent on static shapes (i.e. CUDA graphs). A potential solution involves integrating ‘ghost molecules’, akin to strategies used in static neighbor list shaping, to standardize the atom count inputted to the network. However, this method increases memory consumption in an already memory-constrained environment and raises concerns about the backpropagation of losses for these non-existent atoms, which may lead to numerical instability.

Moreover, training necessitates backpropagation through the network. In our context, this involves a double backpropagation process when the loss function includes force calculations. Currently, double backpropagation is inadequately supported by the PyTorch compiler. A workaround is to manually implement the network’s initial backward pass (specifically, the force computation). This adjustment enables Autograd to perform only a single backward pass during training, leveraging the PyTorch compiler’s capabilities. Nevertheless, challenges persist with the PyTorch compiler when managing dynamic input shapes.

Given the current constraints, the current release does not include any training-specific optimizations besides the improved dataloader support as previously described.

## 3 Results

### 3.1 Validation

In this subsection, we evaluate the impact of the architectural modifications introduced in the models on predictive accuracy. In the case of TensorNet the modifications targeted its computational performance alone, while for the ET one needs to consider the changes induced by vector_cutoff = True.

#### 3.1.1 Accuracy with TensorNet

**Figure 4:**
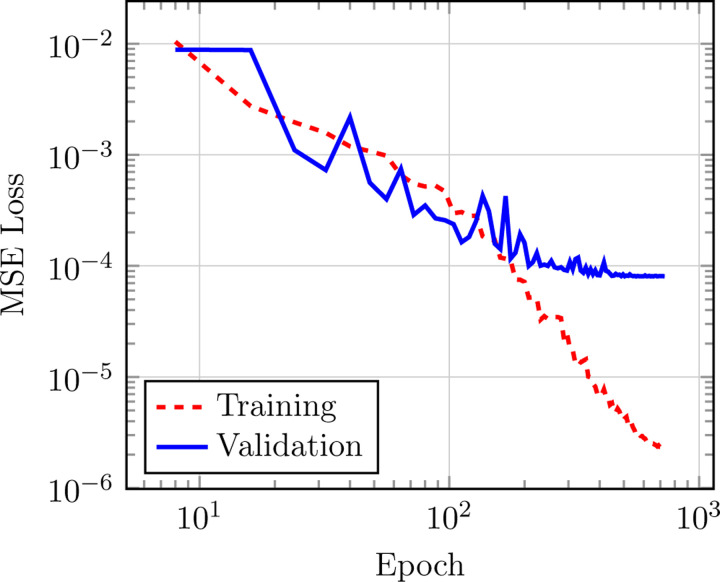
Training and validation curves for TensorNet on the QM9 *U*_0_ benchmark, hyperparameters are in Table 5.

Original test MAE presented in Ref. 13 for the QM9 *U*_0_ target quantity is 3.9(1) meV, while the latest optimized versions of the model (see Figure 4) yield 3.8(2) meV, confirming that the architectural optimizations do not affect TensorNet’s prediction performance. The training loss was computed in this case as the MSE between predicted and true energies. This state-of-the-art performance is achieved with the largest model with 4.0 million trainable parameters, with specific architectural and training hyperparameters being found in Table 5. We also provide in Table 1 the accuracy of smaller and shallower models on the same QM9 quantity (that is, using the same hyperparameters as in Table 5, except for embedding_dimension = 128 and num_layers = 0, 1, 2), while comparing them to other NNPs. Overall, TensorNet demonstrates very satisfactory performances, achieving close to state-of-the-art accuracy (*<* 5 meV MAE) with a very reduced number of parameters.

#### 3.1.2 Accuracy with the Equivariant Transformer

**Figure 5:**
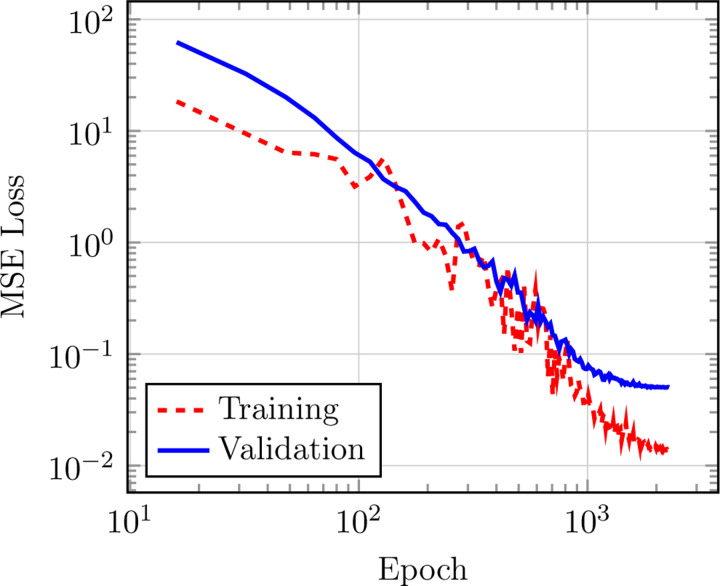
Training and validation curves for the new implementation of the Equivariant Transformer on the MD17 benchmark, hyperparameters are in Table 3.

As previously mentioned, we provide an implementation of the ET where it is modified by applying the cutoff function to the values’ pathway of the attention mechanism to enforce a continuous energy landscape at the cutoff distance. Therefore, we checked to which extent these changes, together with TorchMD-Net’s ones, affect the accuracy of the Equivariant Transformer. We trained the model on the MD17 aspirin dataset (Figure 5) using the hyperparameters defined for the original version of the ET (Table 3, with the addition of vector_cutoff = True), giving final test MAEs of 0.139 kcal/mol and 0.232 kcal/mol/Åin energies and forces, respectively, compared to the original implementation which gave 0.123 kcal/mol and 0.253 kcal/mol/Å.^11^ Regarding QM9 *U*_0_, we reused the original hyperparameters for the dataset found in Table 4 (again, adding vector_cutoff = True), and comparative results can be found in Table 1.

### 3.2 Molecular Simulations

**Figure 6:**
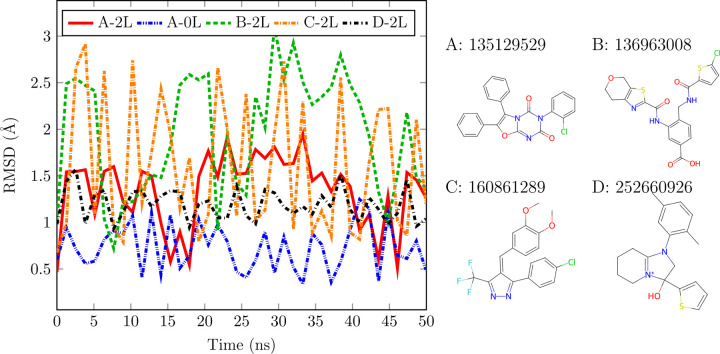
(Left) RMSD analysis for the trajectories of 4 molecules outside of the training set. Simulations are carried out with TensorNet 2L, using the parameters in table 6, with the exception of A-0L, in which a 0L TensorNet model is showcased. Presented data is plotted only every 1.28 ns for visualization clarity. (Right) Representation of the simulated molecules. Labels show the PubChem ID for each molecule.

We performed NVT molecular dynamics simulations employing TensorNet models trained on the ANI-2x dataset.^45^ A table detailing the hyperparameters is provided for reference in Table 6. Note that we did not include any physical priors in these trainings nor in the subsequent simulations, i.e all forces in the system come from the model itself. Starting from the SPICE dataset,^37^ we selected the PubChem subset and utilized it to create a test set comprising four randomly chosen conformers. This test set aimed to evaluate the ability of the NNP to perform stable molecular dynamics (MD) simulations on molecules not encountered during the training stage.^61,62^ The training dataset, as well as the PubChem subset, represent a broad diversification of molecules containing the elements H, C, N, O, S, F, Cl. To generate the input data, the SMILES and the coordinates of interest were used to build a molecule object using openff-toolkit,^63^ and the atomic numbers were used as embeddings. Using the more accurate TensorNet 2L model, a 50 ns trajectory with a time-step of 1 fs was generated for each molecule using OpenMM’s^19^ LangevinMiddleIntegrator at 298.5K and a friction coefficient of 1 ps^*−*1^. We also used for one of the molecules a TensorNet 0L model with the same simulation settings to test its stability. A root mean square displacement (RMSD) analysis was performed for each trajectory taking the starting conformation as reference, see Figure 6. The results highlight the model’s ability to run stable MD simulations, even for the 0L case where the model’s receptive field and parameter count are substantially reduced.

### 3.3 Speed performance

All results presented in this work were carried out using an NVIDIA RTX4090 GPU (driver version 525.147) with a 32 core Intel(R) Xeon(R) Silver 4110 CPU in Ubuntu 20.04. We used CUDA 12.0 with Pytorch version 2.1.0 from conda-forge. We provide all timings in million steps per day, which can be easily converted to nanoseconds per day. These units are more commonly used in molecular dynamics settings, and the conversion can be done by taking the quantity in million steps per day times the timestep in femtoseconds. Therefore, for example, 1 million steps per day is equivalent to 1 ns/day for a timestep of 1 fs.

**Figure 7:**
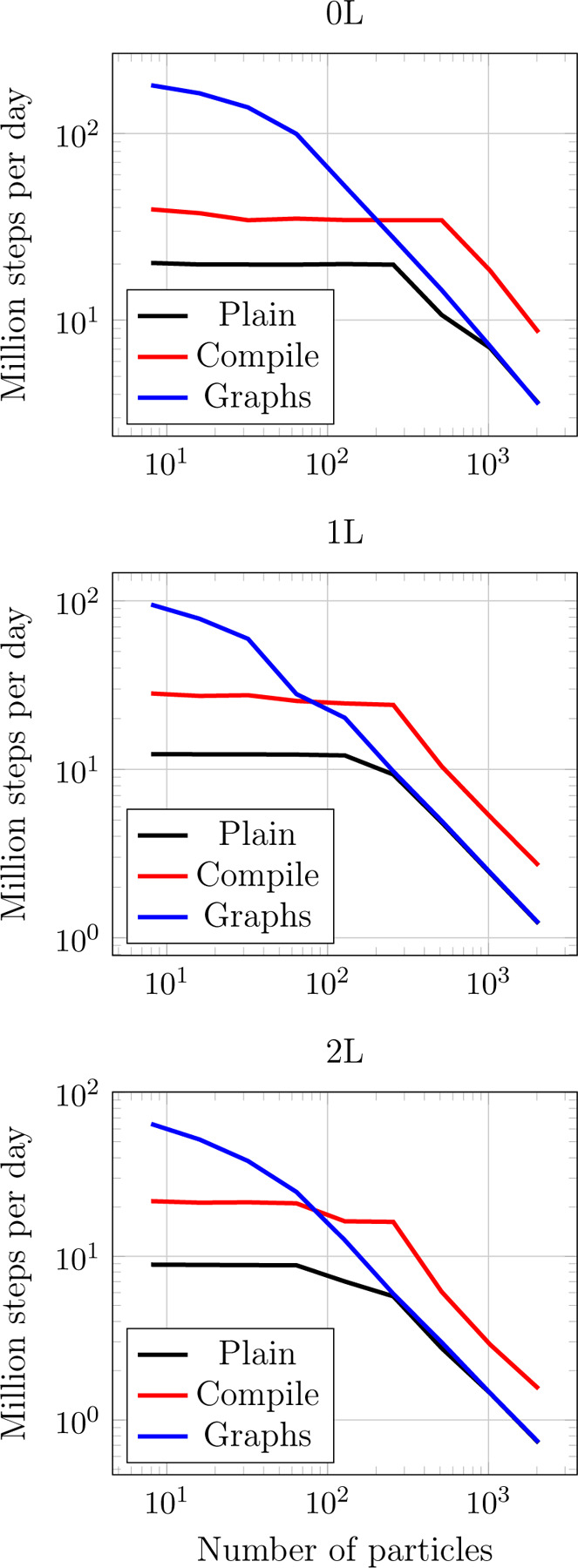
Comparison between TensorNet inference times (energy and forces) with 0, 1 and 2 layers, embedding dimension 128, 64 neighbors on average. All atoms are passed in a single batch. Plain represents the bare TensorNet implementation; with Compile the module has been preprocessed with torch.compile with the “max-autotune” option; for Graphs the whole computation has been captured into a single CUDA graph.

To study the optimization strategies laid out in section 2.5 we show energy and forces inference performance for several equivalent implementations of TensorNet in Figure 7. Note that in TorchMD-Net, running inference requires one backpropagation step to compute forces as the negative gradient of the energies with respect to the input positions, which are computed via Autograd. This step is also included in these benchmarks. We make sure not to include any warmup times in these benchmarks by running the models for 100 iterations before timing. We refer as “Graph” to an implementation that has been modified to ensure every CUDA graph requirement is met. For “Compile” the implementation is carefully tailored to look for the best performance in torch.compile in addition to the changes introduced for “Graph”. Finally, “Plain” represents the baseline implementation in PyTorch.

Although in principle the code received by the compiler is entirely capturable by a graph, it often decides to capture only some sections of it, introducing other kinds of optimizations instead. This is also made evident by the appearance of the same kind of “plateau” performance for smaller workloads in both Plain and Compile, which can be attributed to a bottleneck produced by kernel launch overhead. Still, the torch compiler is able to provide a speedup of a factor 2 to 3 for all workloads with respect to the original implementation.

CUDA kernel overhead (and thus the performance gain of CUDA graphs) is expected to dominate for small workloads, where it is usual for the kernel launching time to be larger than the actual execution. Figure 7 indeed corroborates this by showing speedups between 10 and 2 times for molecules with up to a few hundreds of atoms and for all numbers of interaction layers (0, 1 and 2). Starting from workloads consisting of several hundreds of atoms, the performance of the Plain version is recovered.

We also explore inference times for some molecules with varying number of atoms in Table 2. For these molecules, which can be found in the repository for speed benchmarking purposes, we measure time to compute the potential energy and atomic forces of a single example using TensorNet with 0, 1 and 2 interaction layers. Again, we express this time in million steps per day. In all cases we use a cutoff of 4.5Å, an embedding dimension of 128, 32 radial basis functions and a maximum of 32 neighbors per particle.

## 4 Conclusions

TorchMD-Net has significantly evolved in its recent iterations, becoming a comprehensive platform for neural network potentials (NNPs). It provides researchers with robust tools for both rapid prototyping of new models and executing production-level tasks. However, despite these advancements, NNPs still face substantial challenges before they can fully replace traditional force fields in molecular dynamics simulations. Currently, while the necessary software infrastructure is largely in place, as evidenced by the first-class support for NNPs in popular packages,^19^ issues such as memory requirements and computational performance remain significant concerns.

The impact of memory limitations is anticipated to diminish with ongoing hardware advancements. Yet, enhancing computational performance to a level that is competitive with traditional methods necessitates more intricate strategies. This involves developing architectures and their implementations in a manner that leverages the full capabilities of GPU hardware.

From a software development perspective, the compilation functionality within PyTorch is an evolving feature, still in its early stages. Its current development trajectory, which aims to minimize the necessary code modifications for effective utilization, suggests that future PyTorch releases will likely bring performance enhancements. Continuous improvements in the relevant toolset, encompassing PyTorch, CUDA, Triton, and others, are gradually narrowing the performance gap between highly optimized code and more straightforward implementations.

## Figures and Tables

**Table 1:** Mean absolute error in meV for different models trained on QM9 target property *U*_0_. TensorNet 3L* uses an embedding dimension of 256, while in other cases 128. For the ET, subscripts new and old correspond to the new and the original implementation, that is, with vector_cutoff = True and False, respectively.

Model	*U*_0_ MAE (meV)
Cormorant^55^	22
SEGNN^56^	15
SchNet^57^	14
EGNN^29^	11
Equiformer^58^	6.6
DimeNet++^59^	6.3
SphereNet^60^	6.3
ET_old_^11^	6.2
PaiNN^28^	5.9
Allegro^26^	4.7
MACE^27^	4.1

ET_new_	5.7
TensorNet 0L	7.2
TensorNet 1L	4.7
TensorNet 2L	4.4
TensorNet 3L*	3.9

**Table 2:** TensorNet inference times in million steps per day for the “Plain” (**P**), “Compile” (**C**) and “Graph” (**G**) implementations and varying number of layers.

Molecule (atoms)	P 0L	P 1L	P 2L	C 0L	C 1L	C 2L	G 0L	G 1L	G 2L
Alanine dipeptide (22)	19.86	10.29	8.50	40.19	28.70	21.23	172.80	84.71	56.47
Testosterone (49)	15.05	11.93	8.56	38.57	27.00	21.49	154.29	63.53	39.82
Chignolin (166)	19.77	11.88	7.90	36.77	24.90	21.39	77.14	26.02	15.57
DHFR (2489)	5.56	1.67	0.98	14.47	3.27	1.83	5.65	1.69	1.00
Factor IX (5807)	2.32	0.69	0.41	5.42	1.35	0.77	2.33	0.70	0.42

## A Hyperparameters

In the pursuit of transparency and reproducibility, this appendix provides a detailed account of the hyperparameters employed in our computational experiments. The tables contained herein present the specific values and settings used to achieve the results discussed in the main body of this paper. Readers and fellow researchers are encouraged to refer to these tables when attempting to replicate our results or when utilizing the torchmd-train utility for their own training purposes.

**Table 3:** MD17 hyperparameters used for the ET training in Figure 5.

Parameter	Value
activation	silu
attn_activation	silu
batch_size	8
cutoff_lower	0.0
cutoff_upper	5.0
derivative	True
distance_influence	both
early_stopping_patience	300
ema_alpha_neg_dy	1.0
ema_alpha_y	0.05
embedding_dimension	128
lr	1e-3
lr_factor	0.8
lr_min	1e-7
lr_patience	30
lr_warmup_steps	1000
neg_dy_weight	0.8
num_heads	8
num_layers	6
num_rbf	32
seed	1
train_size	950
val_size	50
vector_cutoff	True
y_weight	0.2

**Table 4:** QM9 *U*_0_ hyperparameters used to obtain the results for ET_**new**_ in Table 1.

Parameter	Value
activation	silu
attn_activation	silu
batch_size	128
cutoff_lower	0.0
cutoff_upper	5.0
derivative	False
distance_influence	both
early_stopping_patience	150
ema_alpha_neg_dy	1.0
ema_alpha_y	1.0
embedding_dimension	256
lr	4e-4
lr_factor	0.8
lr_min	1e-7
lr_patience	15
lr_warmup_steps	10000
neg_dy_weight	0.0
num_heads	8
num_layers	8
num_rbf	64
remove_ref_energy	true
seed	1
train_size	110000
val_size	10000
vector_cutoff	True
y_weight	1.0

**Table 5:** QM9 *U*_0_ hyperparameters used for the trainings in Figure 4 to obtain the results for TensorNet 3L* in Table 1.

Parameter	Value
activation	silu
batch_size	16
cutoff_lower	0.0
cutoff_upper	5.0
Derivative	False
early_stopping_patience	150
embedding_dimension	256
equivariance_invariance_group	O(3)
gradient_clipping	40
lr	1e-4
lr_factor	0.8
lr_min	1e-7
lr_patience	15
lr_warmup_steps	1000
neg_dy_weight	0.0
num_layers	3
num_rbf	64
remove_ref_energy	true
seed	2
train_size	110000
val_size	10000
y_weight	1.0

**Table 6:** ANI2x hyperparameters used for TensorNet training. The model was then used to run the stable molecular dynamics simulations in Figure 6.

Parameter	Value
activation	silu
batch_size	256
cutoff_lower	0.0
cutoff_upper	5.0
derivative	True
early_stopping_patience	50
embedding_dimension	128
equivariance_invariance_group	O(3)
gradient_clipping	100
lr	1e-3
lr_factor	0.5
lr_min	1e-7
lr_patience	4
lr_warmup_steps	1000
neg_dy_weight	100
num_layers	{0,2}
num_rbf	32
seed	1
train_size	0.9
val_size	0.1
y_weight	1.0
